# Lactoferrin is a dynamic protein in human melioidosis and is a TLR4-dependent driver of TNF-α release in *Burkholderia thailandensis* infection in vitro

**DOI:** 10.1371/journal.pntd.0008495

**Published:** 2020-08-07

**Authors:** Shelton W. Wright, Lara Lovelace-Macon, Deirdre Ducken, Sarunporn Tandhavanant, Prapit Teparrukkul, Viriya Hantrakun, Direk Limmathurotsakul, Narisara Chantratita, T. Eoin West

**Affiliations:** 1 Division of Pediatric Critical Care Medicine, Department of Pediatrics, University of Washington, Seattle, Washington, United States of America; 2 Division of Pulmonary, Critical Care and Sleep Medicine, Department of Medicine, University of Washington, Seattle, Washington, United States of America; 3 Department of Microbiology and Immunology, Faculty of Tropical Medicine, Mahidol University, Bangkok, Thailand; 4 Department of Internal Medicine, Sunpasitthiprasong Hospital, Ubon Ratchathani, Thailand; 5 Mahidol-Oxford Tropical Medicine Research Unit, Faculty of Tropical Medicine, Mahidol University, Bangkok, Thailand; 6 Department of Tropical Hygiene, Faculty of Tropical Medicine, Mahidol University, Bangkok, Thailand; University of Texas Medical Branch, UNITED STATES

## Abstract

Melioidosis is an often-severe tropical infection caused by *Burkholderia pseudomallei (Bp*) with high associated morbidity and mortality. *Burkholderia thailandensis* (*Bt*) is a closely related surrogate that does not require BSL-3 conditions for study. Lactoferrin is an iron-binding glycoprotein that can modulate the innate inflammatory response. Here we investigated the impact of lactoferrin on the host immune response in melioidosis. Lactoferrin concentrations were measured in plasma from patients with melioidosis and following ex vivo stimulation of blood from healthy individuals. *Bt* growth was quantified in liquid media in the presence of purified and recombinant human lactoferrin. Differentiated THP-1 cells and human blood monocytes were infected with *Bt* in the presence of purified and recombinant human lactoferrin, and bacterial intracellular replication and cytokine responses (tumor necrosis factor-α (TNF-α), interleukin-1β and interferon-γ) were measured. In a cohort of 49 melioidosis patients, non-survivors to 28 days had significantly higher plasma lactoferrin concentrations compared to survivors (median (interquartile range (IQR)): 326 ng/ml (230–748) vs 144 ng/ml (99–277), p<0.001). In blood stimulated with heat-killed *Bp*, plasma lactoferrin concentration significantly increased compared to unstimulated blood (median (IQR): 424 ng/ml (349–479) vs 130 ng/ml (91–214), respectively; p<0.001). Neither purified nor recombinant human lactoferrin impaired growth of *Bt* in media. Lactoferrin significantly increased TNF-α production by differentiated THP-1 cells and blood monocytes after *Bt* infection. This phenotype was largely abrogated when Toll-like receptor 4 (TLR4) was blocked with a monoclonal antibody. In sum, lactoferrin is produced by blood cells after exposure to *Bp* and lactoferrin concentrations are higher in 28-day survivors in melioidosis. Lactoferrin induces proinflammatory cytokine production after *Bt* infection that may be TLR4 dependent.

## Author summary

Melioidosis is a severe tropical infection caused by the bacterium *Burkholderia pseudomallei*. Despite antibiotics, mortality in some regions remains very high, necessitating the need for alternative treatment strategies, including targeting the immune system. Lactoferrin is an iron-binding protein with a variety of different functions. In this study, we wanted to test whether lactoferrin alters how the immune system responds during melioidosis. To achieve this, we first tested the blood of melioidosis patients and found that patients who later died had higher lactoferrin levels compared to those who survived. We also stimulated blood obtained from healthy individuals with *B*. *pseudomallei* and found that lactoferrin levels increase. We next analyzed whether lactoferrin impaired how the bacteria grows and found that the growth of *Burkholderia thailandensis*, a closely related bacterium, was not affected by the addition of lactoferrin to the media. When human immune cells, called monocytes, were infected with *B*. *thailandensis*, we found that levels of a specific inflammatory protein, TNF-α, increased after adding lactoferrin and that this effect was related to a specific immune recognition pathway called Toll-like receptor 4. These findings provide new data about the role of lactoferrin in modulating the immune response in melioidosis.

## Introduction

Melioidosis is a tropical bacterial infection which can cause pneumonia and sepsis. The etiologic agent of melioidosis, *Burkholderia pseudomallei (Bp)*, a Gram-negative saprophytic bacteria, causes disease following inoculation by inhalation, ingestion or cutaneous exposure [1]. Endemic to southeast Asia and parts of northern Australia, melioidosis has mortality rates upwards of 40% in certain regions, including northeast Thailand, despite appropriate antibiotic therapy [2,3]. Due to the difficulty in eradicating the facultatively intracellular pathogen, treatment regimens last several months [1]. Given its pathogenicity, *Bp* is classified as a Tier 1 select agent by the US Centers for Disease Control and Prevention. Importantly, the worldwide prevalence of melioidosis is unknown and may represent a burgeoning global threat with over 160,000 cases worldwide annually [4].

Lactoferrin is an iron-binding glycoprotein typically found in human milk, saliva and other mucosal secretions and is produced by multiple cells, including neutrophils, during inflammation [5,6]. This apparent enrichment in secretions has been postulated to be related to lactoferrin’s broad antimicrobial properties [7]. Historically, these defense mechanisms were ascribed to lactoferrin’s iron sequestration properties [8,9]. However, more recent evidence suggests that lactoferrin may play a direct antimicrobial role by preventing bacterial biofilm formation, activating proteolysis of bacterial virulence factors and blocking the adhesion of bacteria to host immune cell membranes [6,10]. In fact, lactoferrin’s non-iron binding protein domains have exhibited frequent evolvement of polymorphisms, suggesting an importance of lactoferrin’s other moieties [11]. Taking advantage of these properties, bovine lactoferrin, sharing 77% amino acid homology with human lactoferrin, has been studied as an adjuvant therapy in several clinical trials for patients with sepsis [12,13].

Iron metabolism may play a role in *Bp*’s virulence as iron supplementation enhances bacterial growth in soil and *Bp*-infected mice [14,15]. *Bp* can also capture iron from lactoferrin and transferrin in vivo through a specific siderophore, malleobactin [16]. However, in contrast to several other Gram-negative bacteria, lactoferrin supplementation does not impede *Bp* growth in media [17]. Lactoferrin may also directly regulate innate immunity by interacting with intracellular signaling pathways, including NF-κB [18–20]. The immune response to *Bp* infection is dependent on multiple Toll-like receptor (TLR) pathways, including TLR4 [21]. However, whether lactoferrin regulates the immune response during *Bp* infection is unknown.

We hypothesized that human lactoferrin modulates the innate immune response in melioidosis. We initially tested our hypothesis by measuring plasma lactoferrin in patients with melioidosis as well as *Bp-*stimulated blood of healthy individuals. We then assessed the immunomodulatory effects of lactoferrin experimentally in vitro using *B*. *thailandensis (Bt)*. *Bt* is a closely related bacterium to *Bp* which does not require high containment laboratory conditions but induces a similar innate inflammatory response in vivo and in vitro [22–24].

## Methods and materials

### Ethics statement

The Ethical Review Committee for Research in Human Subjects, Ministry of Public Health, Thailand; the Ethics Committee of the Faculty of Tropical Medicine, Mahidol University, Bangkok, Thailand (MUTM2012-024-01 and MUTM2015-002-01); the Ethical Review Committee for Research in Human Subjects, Sunpasitthiprasong Hospital, Ubon Ratchathani, Thailand (039/2556); the Oxford Tropical Medicine Ethics Committee, Oxford UK (OXTREC172-12); and the University of Washington Human Subjects Division Institutional Review Board (42988) approved the studies involving human subjects research. Written informed consent for enrollment in the clinical studies was obtained from subjects or their representatives at the time of enrollment.

### Melioidosis patient cohort

Subjects aged 18 years or older admitted to Sunpasitthiprasong Hospital, Ubon Ratchathani, Thailand with suspected infection were prospectively enrolled between 2013 through 2017. Inclusion criteria were enrollment within the first 24 hours of admission to the hospital and the presence of at least three documented systemic manifestations of infection, as proposed by the 2012 Surviving Sepsis Campaign [25]. Patients suspected of having a hospital-acquired infection, those hospitalized in the 30 days prior to the current hospitalization or those transferred from another healthcare facility after more than 72 hours were excluded. This cohort, and subsets of it, have previously been described [26–29]. For this study, we analyzed plasma, obtained at the time of enrollment, from the subset of 49 subjects with melioidosis, defined by any growth of *Bp* in any clinical specimen submitted for culture, recruited during the first year of the study. Subjects were followed until 28 days after enrollment to determine survival.

### Healthy donor whole blood stimulation

Three hundred healthy Thai adults presenting to donate blood at Sunpasitthiprasong Hospital, Ubon Ratchathani, Thailand were recruited. After subjects provided informed consent, a peripheral blood sample was obtained and stimulated as described previously [30]. For this study, we analyzed blood stimulated with heat-killed *Bp* and unstimulated blood. Blood was mixed 1:1 with RPMI media and 380μl were added to a pre-prepared 96-well plate containing 20μl heat-killed *Bp* K96243 (to achieve a final concentration of 2.5 x 10^6^ CFU/ml) or 20μl media. Plates were then incubated for 6 hours at 37°C under 5% CO_2_ before being spun down, and plasma was removed and frozen.

### Lactoferrin assay

Frozen plasma samples were thawed and human lactoferrin concentrations measured by ELISA (EMD Millipore, San Diego, CA) according to the manufacturer’s directions. Lactoferrin levels in all samples were within the manufacturer’s stated range of detection.

### Bacterial culture

*B*. *thailandensis* E264 [24] was cultured at 1 x 10^3^ CFU/ml at 37°C in either Luria broth (LB), tryptic soy broth (TSB) or M9, minimal salt, media. LB media was prepared by mixing 20g of Difco LB broth (BD Biosciences, Franklin Lakes, NJ) with 1 liter of water. TSB media was prepared by mixing 30g of TSB broth (Sigma-Aldrich, St. Louis, MO) with 1 liter of water and 50 mM glutamate and 1% glycerol. M9 media was prepared by combining 6g Na_2_HPO_4_, 3g KH_2_PO_4_, 0.5g NaCl, 1g NH_4_Cl, 20 ml 20% glucose, 1ml 1M MgSO_4_, 0.1 ml 1M CaCl_2_ in 1L of water [31]. Partially iron-saturated recombinant human lactoferrin (rhLTF; L4040, 0.07% Fe saturation, expressed in rice, Sigma-Aldrich, St. Louis, MO) or partially saturated purified human lactoferrin (phLTF; L0520, 0.006% Fe saturation, purified from human milk, Sigma-Aldrich) were added to the culture media at varying concentrations. Bacterial colony forming units in the broth were quantified using serial dilutions at 3, 6 and 24 hours after inoculation.

### Cell culture

Human monocytic THP-1 cells were purchased and cultured according to manufacturer specifications (ATCC TIB-202; Manassas, VA). THP-1 cells were cultured in RPMI 1640 with 10% FBS, 1% L-glutamine, 1% HEPES and 0.05 mM 2-mercaptoethanol. For differentiation to macrophages, THP-1 cells were stimulated with 100 nM Vitamin D3 for 24 hours in a 48-well polystyrene plate at a density of 2 x 10^5^ cells/well prior to infection.

Peripheral blood samples were obtained from healthy subjects at Harborview Medical Center, Seattle, USA. Peripheral blood was collected in three 8 ml sodium citrate-containing vacutainers (BD Biosciences, Franklin Lakes, NJ). Peripheral blood monocytes were isolated with centrifugation within 1 hour of collection. Monocyte isolation was performed using a microbead monocyte separation kit (Monocyte Isolation Kit II) according to the manufacturer’s specifications (Miltenyi Biotec, Bergisch Gladbach, Germany). Monocytes were then plated on a 48-well polystyrene plate at 2x10^5^ cells/well, suspended in RPMI 1640 with 10% fetal bovine serum (FBS), 1% L-glutamine and 1% 4-(2-hydroxyethyl)-1-piperazineethanesulfonic acid (HEPES) overnight prior to infection.

### Monocyte infection

Both peripheral blood monocytes and differentiated THP-1 cells were infected with *Bt* at a multiplicity of infection (MOI) of 1 and 10 in 48-well plates. Plates were centrifuged for 10 minutes at 300 x *g* and incubated at 37°C for 6 hours. At 1 hour after infection, rhLTF or phLTF were added at 100 μg/ml or 500 μg/ml. At 6 hours, supernatants were removed and stored at -30°C pending protein measurement. In samples designated for assessing the role of TLR4, 20 μg/ml of monoclonal mouse IgG1 for TLR4 or IgG1 isotype control (InvivoGen, San Diego, CA) were added to cell cultures one hour prior to infection. As a control condition, designated cells were exposed to either 10 or 50 ng/ml of *E*. *coli* K12-derived LPS (Ultrapure LPS-EK, InvivoGen, San Diego, CA). In samples designated for intracellular bacterial measurement, at 1 hour after infection 100 μg/ml of kanamycin was added. At 6 hours after infection, cells were washed with Hank’s balanced salt solution and lysed with 0.1% Triton-X100 (Promega, Madison, WI) and lysed supernatants were plated and incubated at 37°C for 24 hours, at which point colonies were counted. The endotoxin concentration of the rhLTF and phLTF was quantified by a limulus amebocyte lysate assay (Thermo Scientific, Waltham, MA). The final concentration of endotoxin from lactoferrin within each cell culture was determined to be 0.02 endotoxin units/ml for both rhLTF and phLTF conditions. In samples designated for cytokine measurement, uninfected cells were additionally exposed to either media or 100 μg/ml of rhLTF or phLTF, in order to determine effects from direct stimulation by lactoferrin supplementation.

### Cytokine assays

Concentrations of tumor necrosis factor-α (TNF-α), interleukin-1β (IL-1β) and interferon-γ (IFN- γ) were measured in cell supernatants by ELISA according to manufacturer specifications (R&D, Minneapolis, MN). Absorbance of each sample was determined at 450 nm by microplate reader and final cytokine levels were determined using a standard curve for each experiment.

### Statistical analysis

Combined data following a normal distribution are reported as mean ± SD. The Student’s *t*-test or ANOVA was used to compare groups with normally distributed data. Data following a non-normal distribution are presented as medians with interquartile range (IQR). Correlations of non-normal data were assessed by Spearman’s correlation coefficient. Lactoferrin concentrations were log_10_-transformed for statistical analysis. Comparison of means between individuals were performed using the paired t-test and between unpaired groups using an unpaired t-test. Categorical data were analyzed using the chi-square test. Analyses were performed using Stata version 14.2 (College Station, TX) or GraphPad Prism (San Diego, CA). A two-sided P-value ≤ 0.05 was considered to be significant.

## Results

### Blood lactoferrin concentrations are associated with survival in melioidosis and increase after *Bp* exposure ex vivo

**Fig 1:**
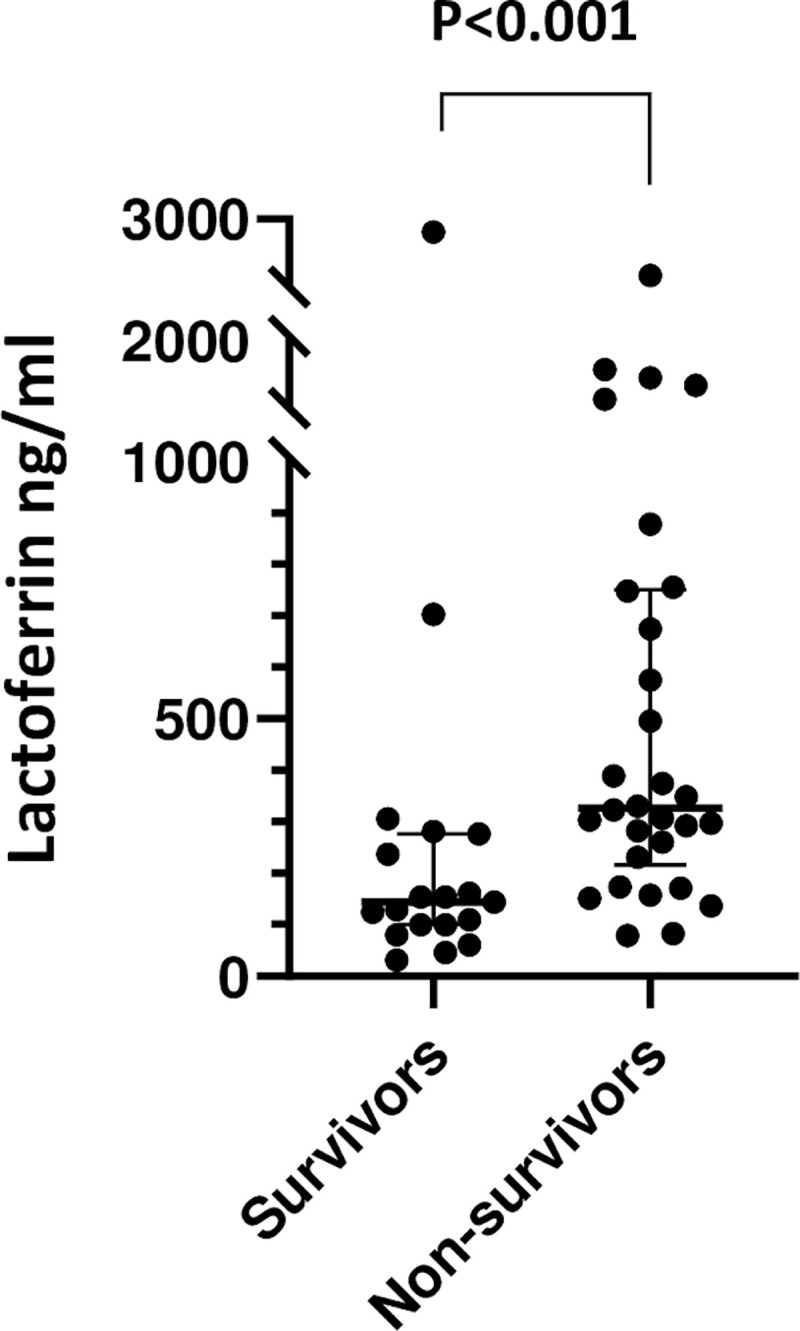
Plasma lactoferrin concentrations are higher in patients who do not survive melioidosis. Plasma lactoferrin concentrations, obtained at study enrollment, in survivors (N = 19) compared to 28-day non-survivors (30) of melioidosis. Data presented as individual values with median and IQR. Comparisons are made following log_10_-transformation of data using unpaired t-test.

To quantify lactoferrin levels in human melioidosis and determine whether they differ based on clinical outcome, we measured plasma lactoferrin concentrations in 49 patients with melioidosis. Subject characteristics are listed in Table 1. Non-survivors to 28 days had significantly higher lactoferrin concentrations compared to survivors (median (IQR): 326 ng/ml (230–748) vs 144 ng/ml (99–277), p<0.001) (Fig 1). As neutrophils can produce lactoferrin, we evaluated whether neutrophil count was correlated with lactoferrin concentration. Neutrophil count was not significantly higher in non-survivors compared to survivors (p = 0.06) and was only moderately correlated with lactoferrin concentration in all subjects with melioidosis (Spearman’s rank correlation coefficient 0.43, p<0.01).

**Table 1:** Melioidosis cohort characteristics.

Characteristics	All (n = 49)	Survivors (N = 19)	Non-survivors (N = 30)
Demographics			
Age, median (IQR)	53 (46–62)	56 (46–70)	51 (42–56)
Male sex, N (%)	30 (61)	14 (74)	16 (53)
Pre-existing conditions			
Charlson Comorbidity Index, median (IQR)	2 (1–3)	1 (1–2)	2 (1–4)
Diabetes, N (%)	21 (43)	7 (37)	14 (47)
Chronic liver disease, N (%)	2 (4)	2 (11)	0
Chronic kidney disease, N (%)	4 (8)	1 (5)	3 (10)
Chronic cardiovascular disease, N (%)	1 (2)	0	1 (3)
Chronic lung disease, N (%)	5 (10)	1 (5)	4 (13)
Cancer, N (%)	1 (2)	0	1 (3)
HIV, N (%)	0	0	0
White blood cell count (*10^3^), median (IQR)^a^	14 (11–18)	14 (11–16)	16 (12–19)
Neutrophil count (*10^3^), median (IQR)^a^	12 (9–15)	11 (8–12)	14 (10–17)^b^
Bacteremia, N (%)	41 (84)	13 (68)	28 (93)

^a^Highest recorded cell count within 24 hours of admission

^b^p = 0.06 for comparison of the neutrophil count in survivors vs. non-survivors

**Fig 2:**
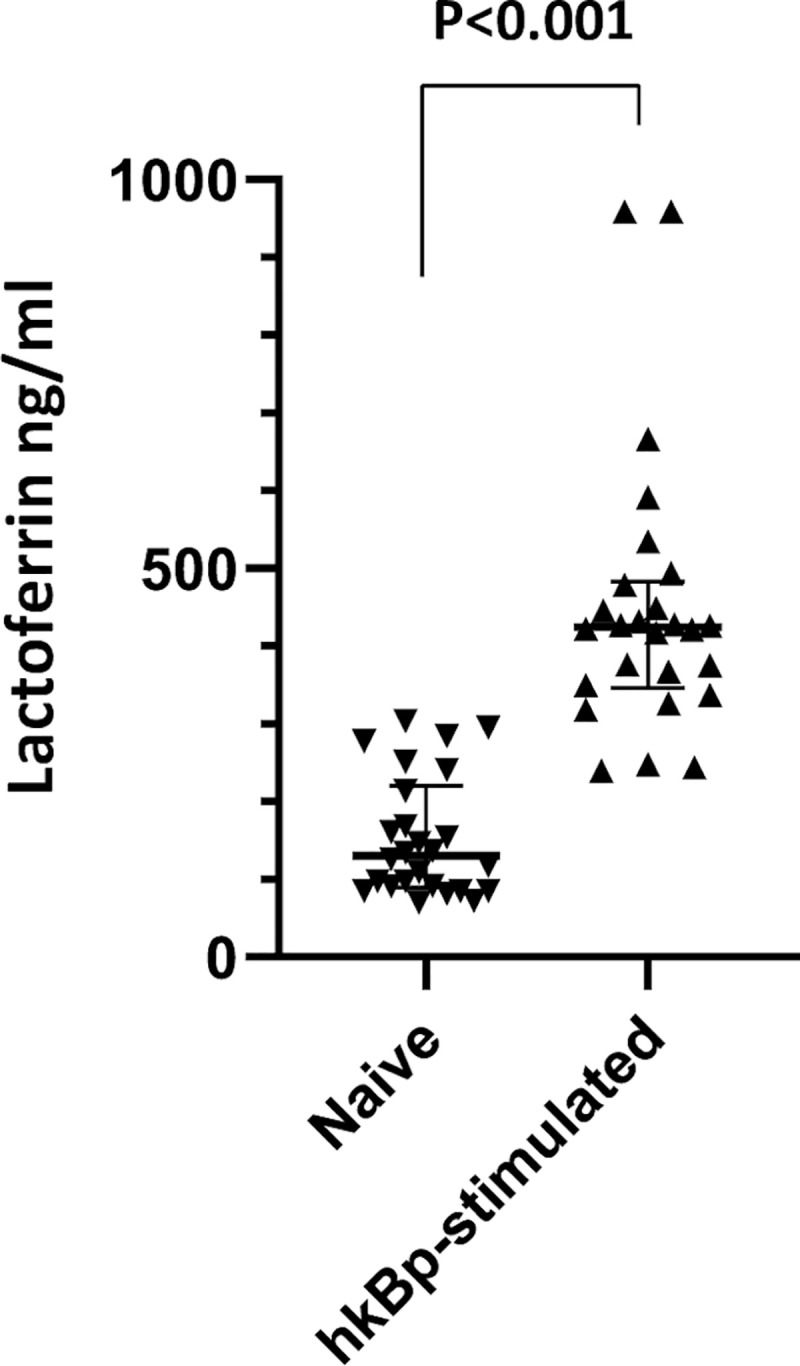
Lactoferrin is produced by blood cells of healthy individuals after stimulation with *Bp*. Plasma lactoferrin concentrations of matched, unstimulated (naïve) blood from healthy individuals and blood stimulated with heat-killed *Bp* K96243 (hkBp) at 2.5 x 10^6^ CFU/ml (N = 26). Data presented as individual values with median and IQR. Comparisons are made following log_10_-transformation of data using paired t-test.

To evaluate whether blood cells produce lactoferrin in response to *Bp*, we compared lactoferrin concentrations in whole blood from 26 healthy individuals stimulated ex vivo with heat-killed *Bp* to concentrations in unstimulated blood from the same individuals. The average lactoferrin concentration in *Bp*-stimulated blood was significantly higher compared to the average concentration in unstimulated blood (median (IQR): 424 ng/ml (349–479) vs 130 ng/ml (91–214), respectively; p<0.001) (Fig 2). The median neutrophil count in the healthy subject cohort was 3,000 with an IQR of 2,200–3,600. Neutrophil count was not correlated with lactoferrin concentration before stimulation (Spearman’s rank correlation coefficient 0.12, p = 0.33) but was more strongly correlated after stimulation (Spearman’s rank correlation coefficient 0.67, p<0.01). These results taken together suggest that early plasma lactoferrin levels are increased in melioidosis patients who do not survive to 28 days, and that *Bp* induces lactoferrin production by blood cells.

### Lactoferrin does not impair *Bt* growth in culture

**Fig 3:**
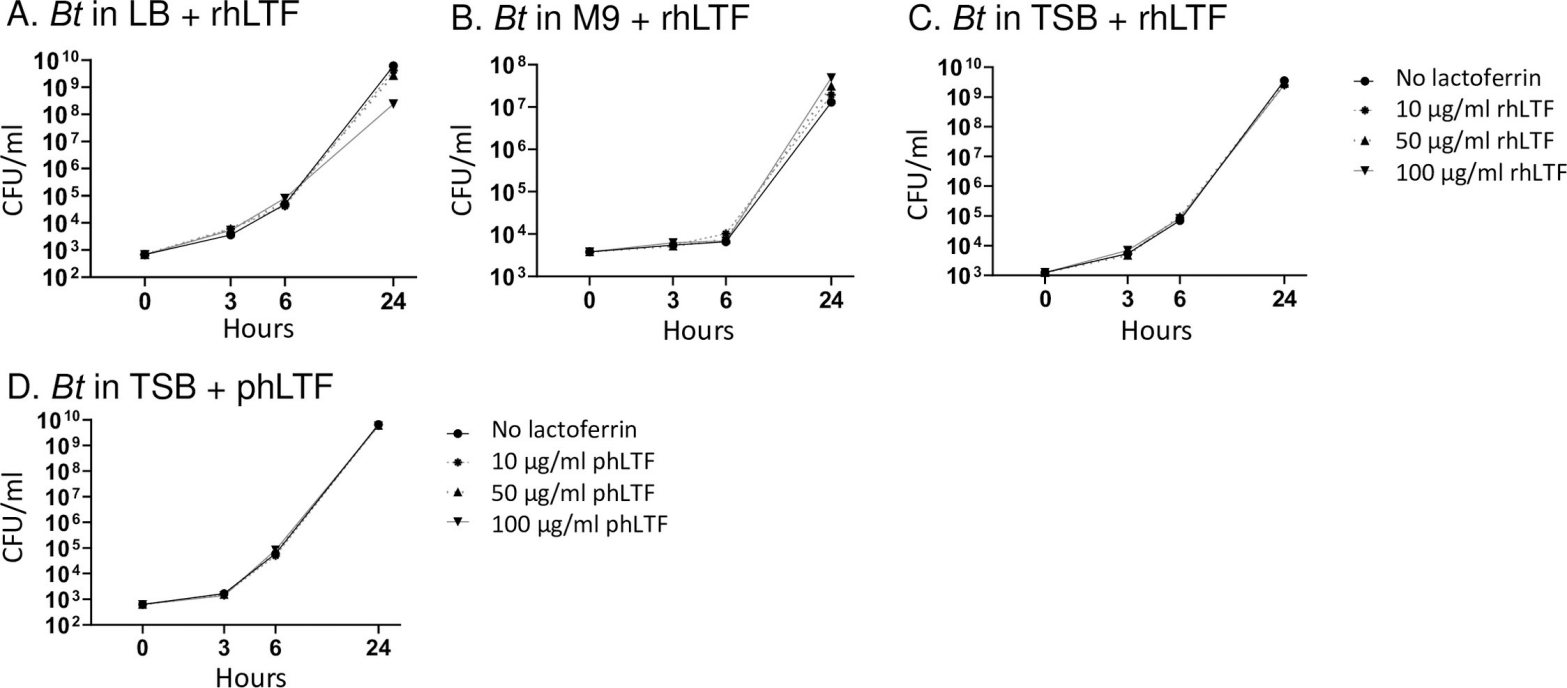
Lactoferrin does not impair growth of *B*. *thailandensis* in media. In the presence of varying concentrations of rhLTF, *B*. *thailandensis* (*Bt*) growth was quantified over 24 hours in A) LB, B) M9 or C) TSB media. In the presence of varying concentrations of phLTF, *Bt* growth was quantified over 24 hours in D) TSB media. Each graph is representative of at least two independent experiments.

Lactoferrin has iron-sequestering capabilities that inhibit bacterial growth as well as microbicidal effects against some bacteria [32]. In order to evaluate whether lactoferrin impairs bacterial growth, *Bt* was cultured in liquid media in the presence of rhLTF. To account for lactoferrin’s ability to sequester iron, three different medias were used: LB, minimal M9 and TSB media. For each of the three medias used, the addition of rhLTF up to 100 μg/ml did not consistently impair growth of *Bt* over 24 hours (Fig 3A–3C). In selected experiments, rhLTF concentrations of 500–1000 μg/ml did not impair *Bt* growth in any media. Similarly, the addition of phLTF (Fig 3D) showed no reduction in *Bt* growth in TSB media at 24 hours.

### Lactoferrin does not inhibit intracellular replication of *Bt* in differentiated THP-1 cells or blood monocytes

**Fig 4:**
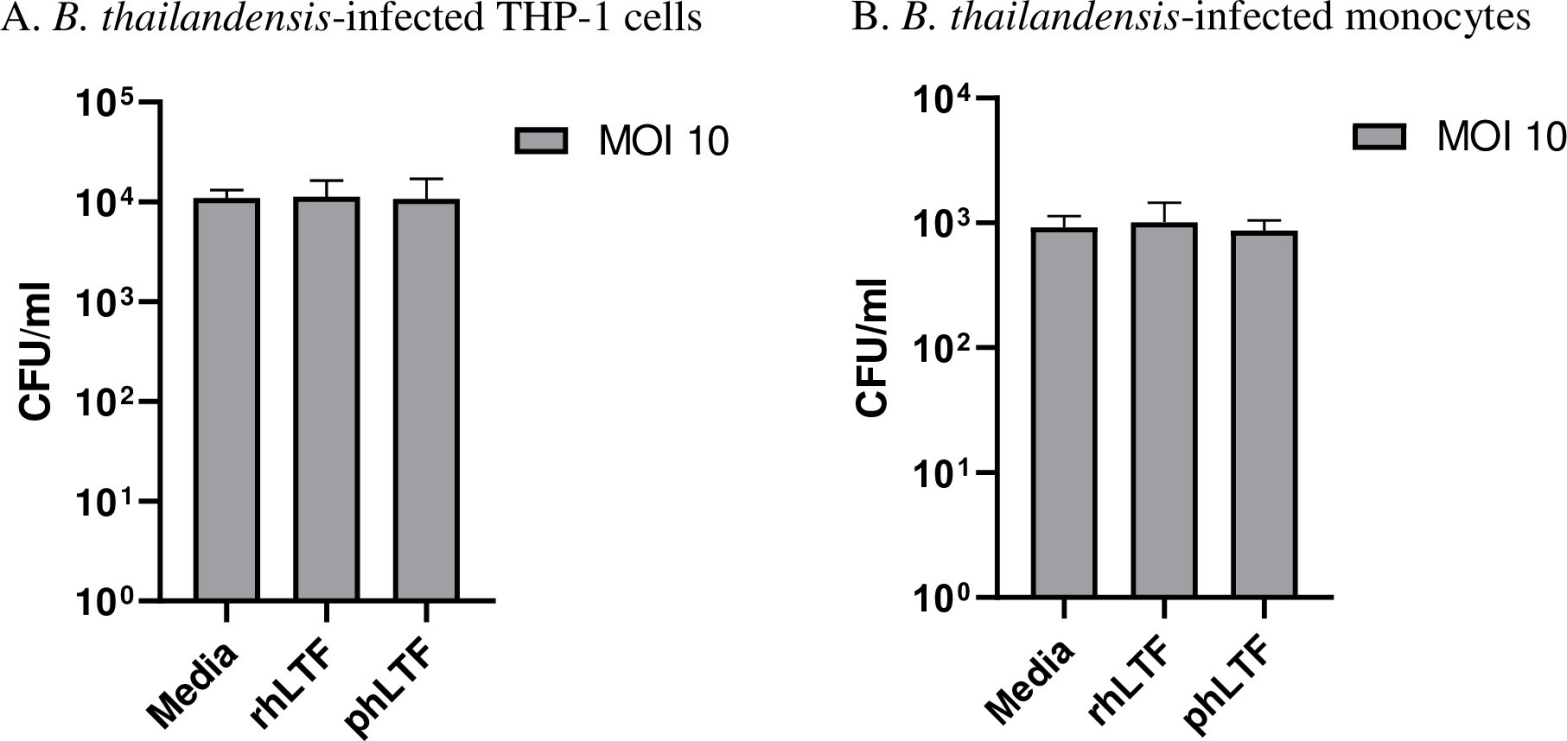
Lactoferrin does not alter intracellular replication of *Bt* in differentiated THP-1 cells or blood monocytes. Differentiated THP-1 cells (A) and primary blood monocytes (B) were infected with *Bt* at an MOI of 10 and 1 hour later were treated with either media or 100 μg/ml of rhLTF or phLTF, along with kanamycin (100 μg/ml). After 6 hours, cells were washed, lysed and intracellular bacteria were cultured and quantified in CFU/ml at 24 hours. Means ± standard deviations of duplicate or triplicate conditions are displayed. rhLTF vs media and phLTF vs media groups were compared by t-test (p>0.05 for all comparisons). One representative example of two or three independent experiments is shown.

*Bp* and *Bt* are facultative intracellular pathogens. This intracellular lifestyle may be an important contributor to subversion or avoidance of the host response and eradication of the bacterium [33]. To determine whether lactoferrin alters intracellular bacterial replication in monocytic cells, we infected differentiated THP-1 cells with *Bt* at an MOI of 10. After one hour either 100 μg/ml of rhLTF or phLTF was added and an antibiotic protection assay was performed. Six hours after infection, no differences in intracellular *Bt* counts were noted between cells exposed to 100 μg/ml of either rhLTF or phLTF compared to control cells (Fig 4A). We repeated this experiment infecting fresh human peripheral blood monocytes with *Bt* at an MOI of 10. We observed no difference in the six-hour intracellular *Bt* counts between cells treated with rhLTF, phLTF or control cells (Fig 4B). Therefore, supplemental lactoferrin does not alter intracellular replication of *Bt* in differentiated THP-1 cells or primary blood monocytes.

### Lactoferrin increases TNF-α production by monocytic cells infected with *Bt*

**Fig 5:**
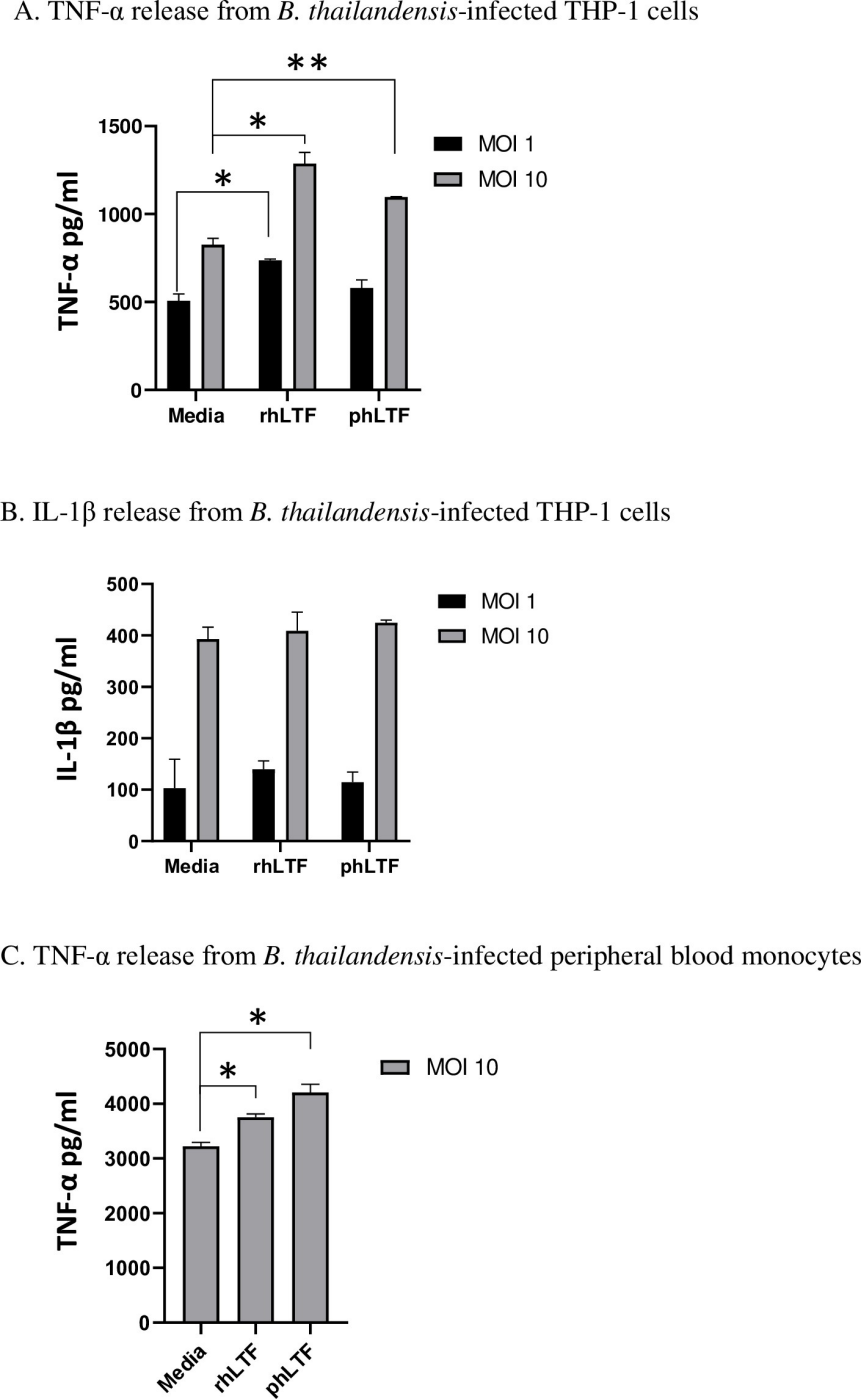
Lactoferrin increases proinflammatory cytokine production by differentiated THP-1 cells or blood monocytes infected with *Bt*. Differentiated THP-1 cells (A-B) and primary blood monocytes (C) were infected with *Bt* at an MOI of 1 (THP-1 cells) and an MOI of 10 (THP-1 cells and primary blood monocytes), and 1 hour later were treated with either media, or 100 μg/ml of rhLTF or phLTF. At 6 hours after infection, cell supernatants were collected and TNF-α (A and C) or IL-1β (B) were measured by ELISA. Means ± standard deviations of duplicate or triplicate conditions are displayed. rhLTF vs media and phLTF vs media groups were compared for each MOI by t-test (* p<0.05; ** p<0.01); The concentrations of cytokines in uninfected THP-1 cells, for all conditions, were below the level of detection. One representative example of two or three independent experiments is shown.

To test whether lactoferrin modulates monocytic cell cytokine production in response to *Bt* infection, differentiated THP-1 cells were infected with *Bt* at an MOI of 1 or an MOI of 10. After one hour, cells were treated with either rhLTF or phLTF. Levels of TNF-α, IL-1β and IFN-γ in cell supernatants were measured by ELISA at 6 hours. At an MOI of 1, infected differentiated THP-1 cells exposed to rhLTF produced significantly more TNF-α compared to infected cells without lactoferrin (p = 0.01; Fig 5A). At an MOI of 10, infected differentiated THP-1 cells exposed to rhLTF or phLTF produced significantly more TNF-α compared to unexposed infected cells (p = 0.01, p = 0.008, respectively; Fig 5A). This effect did not appear to be related to direct stimulation from either rhLTF or phLTF as uninfected cells exposed to either media, rhLTF or phLTF had TNF-α concentrations below the level of detection for the assay. No differences in IL-1β (Fig 5B) or IFN-γ expression (S1 Fig) were noted following differentiated THP-1 cell infection.

The effect of lactoferrin on cytokine production was next tested in human primary blood monocytes infected with *Bt*. Similar to the response noted in infected differentiated THP-1 cells, blood monocytes infected with *Bt* at MOI 10 produced significantly more TNF-α when exposed to either rhLTF (p = 0.02) or phLTF (p = 0.01) compared to no lactoferrin (Fig 5C). While uninfected peripheral blood monocytes had low detectable levels of TNF-α, no difference in concentration was noted between cells exposed to rhLTF or phLTF (S2 Fig). Together, these observations indicate that in monocytic cells infected with *Bt*, lactoferrin promotes TNF-α production.

### The effect of lactoferrin on TNF-α is partially dependent on TLR4 in *Bt* monocyte infection

**Fig 6:**
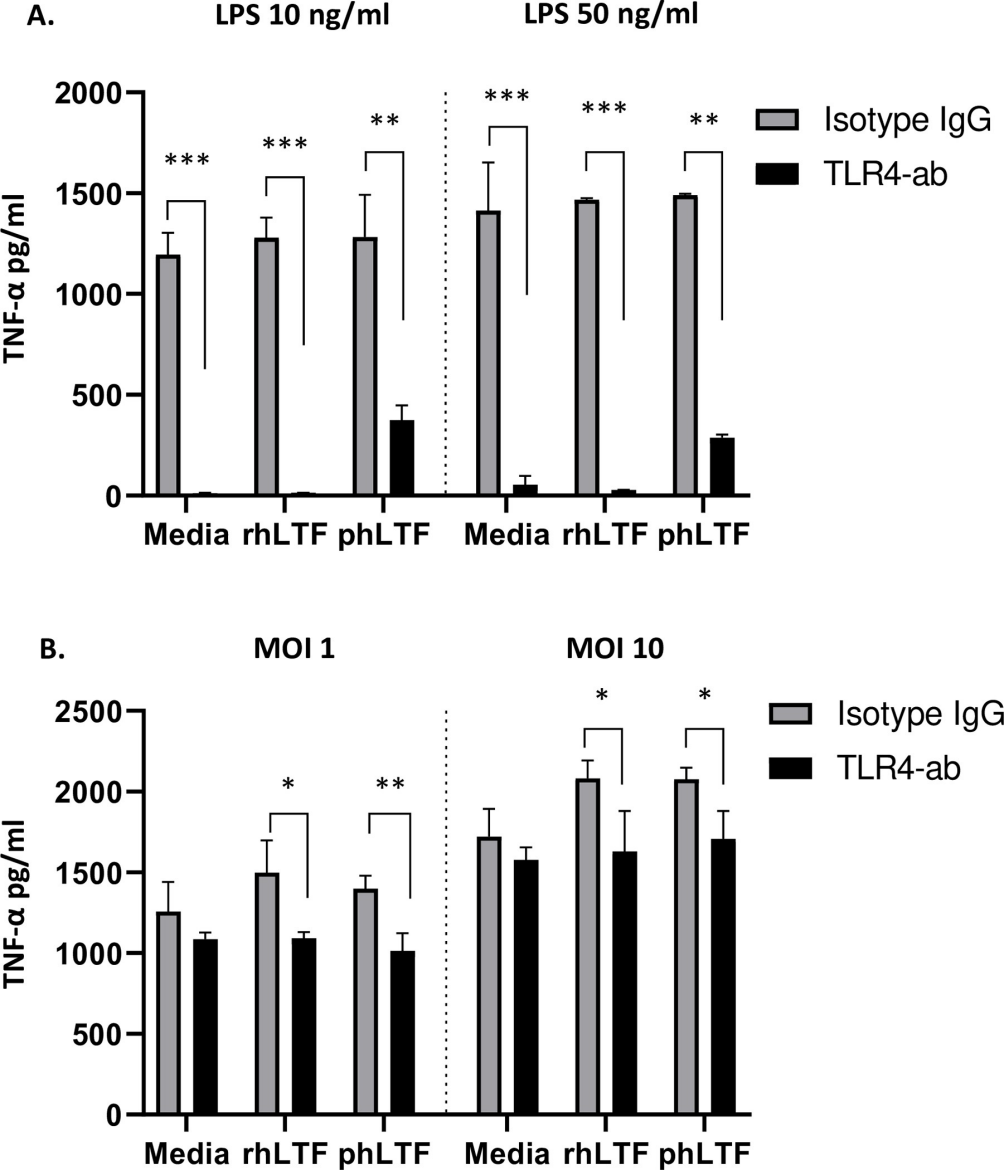
Lactoferrin-induced TNF-α production following *Bt* infection of differentiated THP-1 cells is attenuated by blockade of TLR4. Differentiated THP-1 cells were exposed to *E*. *coli* K12 LPS at 10 ng/ml or 50 ng/ml (A) or infected with *Bt* at an MOI of 1 and 10 (B). At one hour, cells were treated with media or with 100 μg/ml of either rhLTF or phLTF and concurrently with either 20 μg/ml of TLR4-ab or IgG isotype control. After six hours, cell supernatants were collected and TNF-α was measured by ELISA. Means ± standard deviations of duplicate or triplicate conditions are displayed. TLR4-ab vs isotype groups were compared for each condition by t-test (* p<0.05; ** p<0.01; ***p<0.001). The concentration of cytokines in uninfected cells, for all conditions, was below the level of detection. One representative example of two independent experiments is shown.

Lactoferrin may modulate LPS-TLR4 pathways by direct binding of LPS and by nuclear inhibition of NF-κB activation [18,19,34,35]. However, the lactoferrin-LPS complex may also be proinflammatory under certain conditions [36]. In order to determine whether lactoferrin-induced activation of TLR4 may be responsible for the increased TNF-α production by monocytic cells noted in the presence of lactoferrin, we investigated whether the addition of a TLR4-blocking antibody (TLR4-ab) attenuates production of this cytokine. Differentiated THP-1 cells were exposed to either 20 μg/ml of TLR4-ab or an IgG isotype control prior to infection by *Bt* and subsequent treatment with lactoferrin. In control experiments, the addition of TLR4-ab markedly reduced TNF-α production after differentiated THP-1 cells were exposed to *E*. *coli* K12-derived LPS, with or without rhLTF or phLTF treatment (Fig 6A). Following infection of differentiated THP-1 cells with *Bt* at an MOI of 1, treatment with TLR4-ab–compared to an isotype control–significantly reduced supernatant TNF-α concentrations in the presence of rhLTF (p = 0.03) or phLTF (p = 0.008), but not media alone (Fig 6B). A comparable effect was noted for *Bt* infection at an MOI of 10 (Fig 6B). The TLR4-ab-dependent reduction in TNF-α after lactoferrin treatment approximated the baseline TNF-α levels in infected cells not treated with lactoferrin. These results suggest that TLR4 contributes substantially to the lactoferrin-dependent induction of TNF-α production during *Bt* infection.

## Discussion

Melioidosis remains an important cause of sepsis and death in subtropical regions [2] and elucidating the immune response to *Bp* may facilitate the development of novel therapeutics. The main findings of our study are that lactoferrin concentrations are increased in 28-day non-survivors with melioidosis compared to survivors and that lactoferrin concentrations in blood from healthy subjects increase following ex vivo stimulation with heat-killed *Bp*. Furthermore, while lactoferrin does not impair *Bt* growth in culture or intracellular bacterial replication within monocytic cells, lactoferrin drives TNF-α production in *Bt*-infected monocytic cells in a TLR4-dependent manner.

Iron sequestration is likely one mechanism by which lactoferrin exerts antimicrobial properties, including in *Bp* infection [15,16]. However, few reports describe or discern between iron-saturation levels when assessing lactoferrin’s protective effects during bacterial infection. Furthermore, significant variance exists regarding the utilization of human or bovine and recombinant or purified lactoferrin. Here, we specifically analyzed the effect of human lactoferrin, utilizing both recombinant and human milk-purified versions. Iron-saturated lactoferrin–also known as holo-lactoferrin–may become quickly partially unsaturated and quantifying saturation levels can be challenging [37,38]. Our objective was to study lactoferrin-dependent immune modulation rather than antimicrobial properties. In order to limit possible confounding of our results by lactoferrin-dependent iron sequestration, we chose to use partially-iron saturated lactoferrin in our studies after establishing that partially-iron saturated lactoferrin does not impair *Bt* replication. Others have found similarly little effect of supplemental lactoferrin on the growth of *Bp* in culture [17]. However, the degree of iron saturation of rhLTF and phLTF may differ in culture and so this report does not directly address the role of iron saturation on the immunoregulatory properties of lactoferrin.

One of the pathogenic mechanisms of *Bp* and *Bt* is to invade intracellularly, replicate, and ultimately lyse the host cell or infect nearby cells [39–41]. The spread of *Bp* to adjacent host cells results in the formation of multinuclear giant cells which eventually lyse resulting in localized spread [33,42]. *Bp* has variable sensitivity to two potentially bactericidal amino acid residues on bovine lactoferrin [43–45]. Bovine lactoferrin has also been previously described as enhancing phagocytic killing in *Staphylococcus aureus* infection but recently was reported not to interfere with monocytic bacterial uptake [46,47]. In our studies, we found no difference in intracellular bacteria replication when *Bt*-infected, differentiated THP-1 cells were exposed to lactoferrin. These findings suggest that lactoferrin neither enhances, nor interferes with, the intracellular replication of *Bt*.

The initial inflammatory response to *Bp* is characterized by a robust activation of the innate immune system [48–50]. This activation relies, in part, on multiple Toll-like receptor (TLR) pathways, including TLR4 and TLR5 [21,51,52]. Recent population genetic studies have identified TLR genetic variants associated with outcomes in melioidosis, further suggesting an important role of these pathways [51,53]. Besides iron-sequestration and other direct antimicrobial properties, lactoferrin may have immunomodulatory properties as well, though its role in TLR4 signaling is unclear. For example, human lactoferrin may promote TLR4-mediated activation through its carbohydrate chains [35]. Lactoferrin can also bind and sequester LPS or even inhibit LPS-binding, decreasing TLR4 intracellular signaling [54–56]. Conversely, neonatal and adult human monocyte-derived macrophages, differentiated in the presence of lactoferrin, express reduced TLR4 activation and signaling, even after LPS stimulation [57]. Our findings support a TLR4-mediated mechanism of increased TNF-α production in the presence of lactoferrin.

Even in the absence of LPS, lactoferrin increases IL-8 and TNF-α mRNA expression in monocytes [58]. However, lactoferrin may alter LPS-induced intracellular cytokine production through NF-κB interference [18]. In vitro studies of methicillin-resistant *Staphylococcus aureus* (MRSA)-infected monocytes treated with lactoferrin demonstrated an increase in IFN-γ and IL-2 but a decrease in TNF-α, IL-1β and IL-6 [59]. TLR4 activates NF-κB, but so do multiple other pathways, including NF-κB-inducing kinase (NIK). Although lactoferrin does not have a known receptor on monocytes, it can stimulate NIK porcine monocyte-derived macrophages [58]. Lactoferrin can also activate NF-κB through TLR4-independent pathways as well, potentially accounting for the partial attenuation of lactoferrin’s TNF-α production by TLR4-blockade in our study [60].

Our study has several limitations. The association we observed between lower lactoferrin levels and survival in melioidosis patients was not adjusted for potential confounders. Our in vitro experiments may not represent the effects of lactoferrin in a systemic infection model. We also cannot rule out an effect related to the iron-saturation state of our lactoferrin preparations. Complete iron-depletion of lactoferrin is difficult, requiring acidic buffers which may affect protein function [37]. Finally, many other reports regarding the effects of lactoferrin on host response to bacterial infection have used bovine lactoferrin [10,46,58,61]. In our report, we limited our assessment to human-derived lactoferrin and so comparisons are limited. Finally, our infection model utilized *Bt*, a BSL-1 organism, as a surrogate for *Bp* infection. While monocyte infection with *Bt* may yield similar in vitro inflammatory cytokine patterns, further studies are needed to confirm a similar pattern in *Bp* infection [23]. Importantly, we studied the role of lactoferrin on monocyte infection; other cells, including neutrophils, may play a critical role in lactoferrin production during infection [62]. Finally, while we chose to focus on the TLR4 pathway due to its importance in melioidosis, other TLR pathways, including TLR2, may be modulated by lactoferrin [63,64].

In conclusion, our results implicate lactoferrin as a dynamic protein in melioidosis. We show that lactoferrin does not impair growth of *Bt* in culture or intracellular replication in monocytic cells but that it does enhance activation of specific proinflammatory pathways in monocytic cells. Furthermore, we have identified TLR4 as a contributor to this proinflammatory cytokine activation. These investigations add to our growing knowledge of host defense in melioidosis.

## Supporting information

S1 Fig Lactoferrin does not alter IFN-γ production by THP-1 cells infected with *Bt*. THP-1 cells were infected with *Bt* at an MOI of 1 and 10, and 1 hour later were treated with either media, or 100 μg/ml of rhLTF or phLTF. At 6 hours after infection, cell supernatants were collected and IFN-γ was measured by ELISA. Means ± standard deviations of duplicate conditions are displayed. rhLTF vs media and phLTF vs media groups were compared for each MOI by t-test. The concentrations of cytokines in uninfected cells, for all conditions, were below the level of detection. One representative example of two or three independent experiments is shown.
(TIF)

S2 Fig Lactoferrin does not alter TNF-α production by uninfected peripheral blood monocytes. Peripheral blood monocytes were treated with either media, 100 μg/ml of rhLTF, or 100 μg/ml of phLTF. At 6 hours after treatment, cell supernatants were collected and TNF-α was measured by ELISA. Means ± standard deviations of triplicate conditions are displayed. rhLTF vs media and phLTF vs media groups were compared by t-test and both comparisons were above the limit of statistical significance (p>0.3 for both). One representative example of two independent experiments is shown.
(TIF)
