# Supplementary figures and images for: Lactoferrin is a dynamic protein in human melioidosis and is a TLR4-dependent driver of TNF-α release in Burkholderia thailandensis infection in vitro
Source: PLoS Negl Trop Dis. 2020 Aug 7;14(8):e0008495. doi: 10.1371/journal.pntd.0008495 (PMC7439809; doi:10.1371/journal.pntd.0008495)

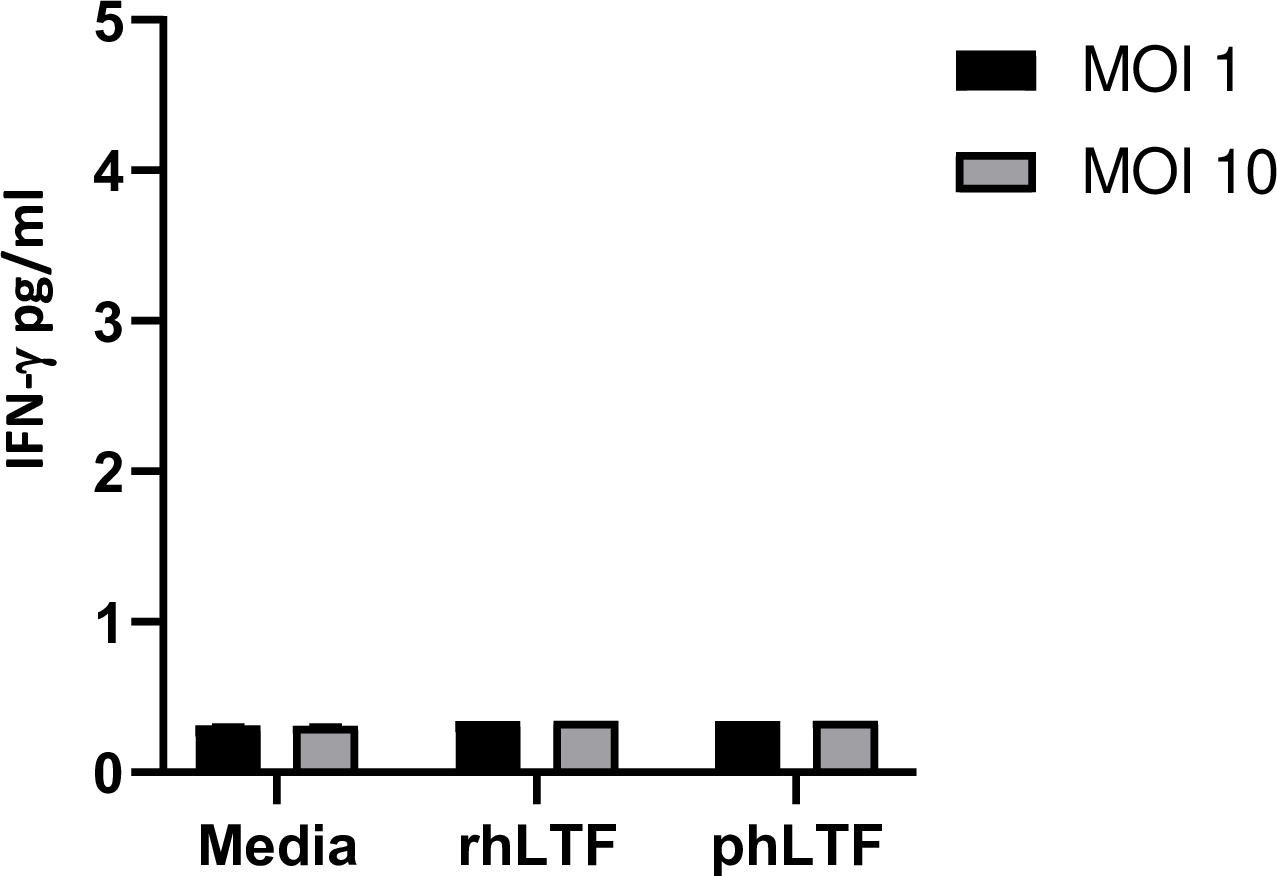

Supplement: S1 Fig — THP-1 cells were infected with Bt at an MOI of 1 and 10, and 1 hour later were treated with either media, or 100 μg/ml of rhLTF or phLTF. At 6 hours after infection, cell supernatants were collected and IFN-γ was measured by ELISA. Means ± standard deviations of duplicate conditions are displayed. rhLTF vs media and phLTF vs media groups were compared for each MOI by t-test. The concentrations of cytokines in uninfected cells, for all conditions, were below the level of detection. One representative example of two or three independent experiments is shown. (TIF) [file pntd.0008495.s001.tif]

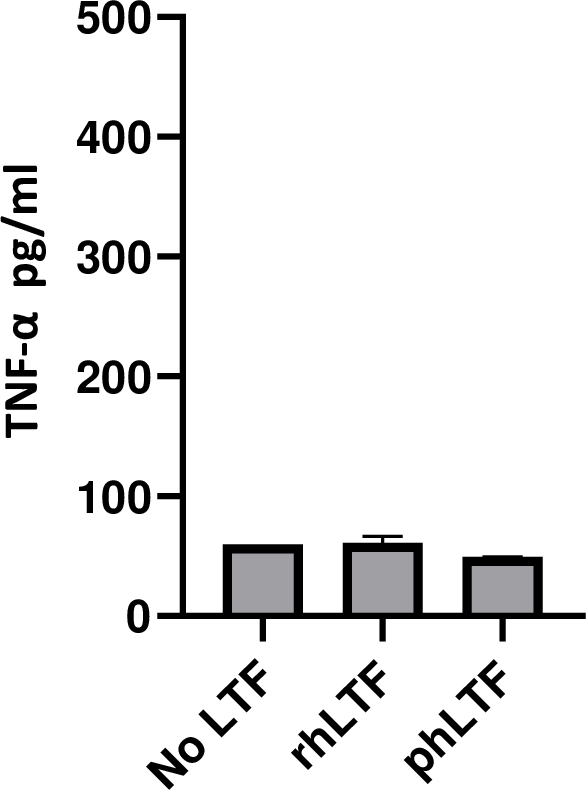

Supplement: S2 Fig — Peripheral blood monocytes were treated with either media, 100 μg/ml of rhLTF, or 100 μg/ml of phLTF. At 6 hours after treatment, cell supernatants were collected and TNF-α was measured by ELISA. Means ± standard deviations of triplicate conditions are displayed. rhLTF vs media and phLTF vs media groups were compared by t-test and both comparisons were above the limit of statistical significance (p>0.3 for both). One representative example of two independent experiments is shown. (TIF) [file pntd.0008495.s002.tif]
